# Acute myocardial infarction with simultaneous total occlusion of the left anterior descending artery and right coronary artery successfully treated with percutaneous coronary intervention

**DOI:** 10.1186/s12872-022-02652-3

**Published:** 2022-05-10

**Authors:** Ryuhei Saito, Kohei Koyama, Ken Kongoji, Kyoko Soejima

**Affiliations:** Department of Cardiology, Kyorin University School of Medicine, 6-20-2 Shinkawa, Mitaka City, Tokyo 181-8611 Japan

**Keywords:** Acute myocardial infarction, Simultaneous total occlusion, Impella CP

## Abstract

**Background:**

Simultaneous thrombosis in more than one coronary artery is an uncommon angiographic finding in patients with acute ST-segment elevation myocardial infarction. It is difficult to identify using 12-lead electrocardiography and usually leads to cardiogenic shock and fatal outcomes, including sudden cardiac death. Therefore, immediate revascularization and adequate mechanical circulatory support are required.

**Case presentation:**

We report the case of a 58-year-old man who presented with vomiting and chest pain complicated by cardiogenic shock and complete atrioventricular block. Electrocardiography revealed ST-segment elevation in leads II, III, aVF, and V1–V6. Emergency coronary angiography revealed total occlusion of the proximal left anterior descending artery and right coronary artery. The patient successfully underwent primary percutaneous coronary intervention with ballooning and stenting for both arteries. An Impella CP was inserted during the procedure. Fifty-seven days after admission, he had New York Heart Association class II heart failure and was transferred to a rehabilitation hospital.

**Conclusions:**

Acute double-vessel coronary thrombosis, a serious event with a high mortality rate, requires prompt diagnosis and management to prevent complications such as cardiogenic shock and ventricular arrhythmias. A combination of judicious medical treatment, efficient primary percutaneous coronary intervention, and early mechanical support device insertion is crucial to improve the survival rate of patients with this disease.

**Supplementary Information:**

The online version contains supplementary material available at 10.1186/s12872-022-02652-3.

## Background

Coronary plaque rupture and acute thrombosis are the primary mechanisms of acute myocardial infarction (AMI). ST-elevation myocardial infarction (STEMI) is usually caused by acute thrombosis of only one epicardial artery, which is termed the culprit vessel [1, 2]. STEMI caused by the simultaneous thrombosis of multiple coronary arteries is rare and difficult to identify using 12-lead electrocardiography (ECG). AMI due to simultaneous total occlusion of multiple coronary arteries, including the left main coronary artery, is rare. Although multiple cases of simultaneous coronary occlusion have been reported and various mechanisms have been postulated, the exact mechanism remains unclear. It is a life-threatening situation associated with rapid deterioration of left ventricular function, often leading to cardiogenic shock, and ultimately, death. Therefore, immediate revascularization is necessary. Here, we present the case of a patient with simultaneous total occlusion of the two main coronary arteries who was treated with immediate complete revascularization and implantation of an Impella device and in whom we observed a relatively good clinical course.

## Case presentation

**Fig. 1:**
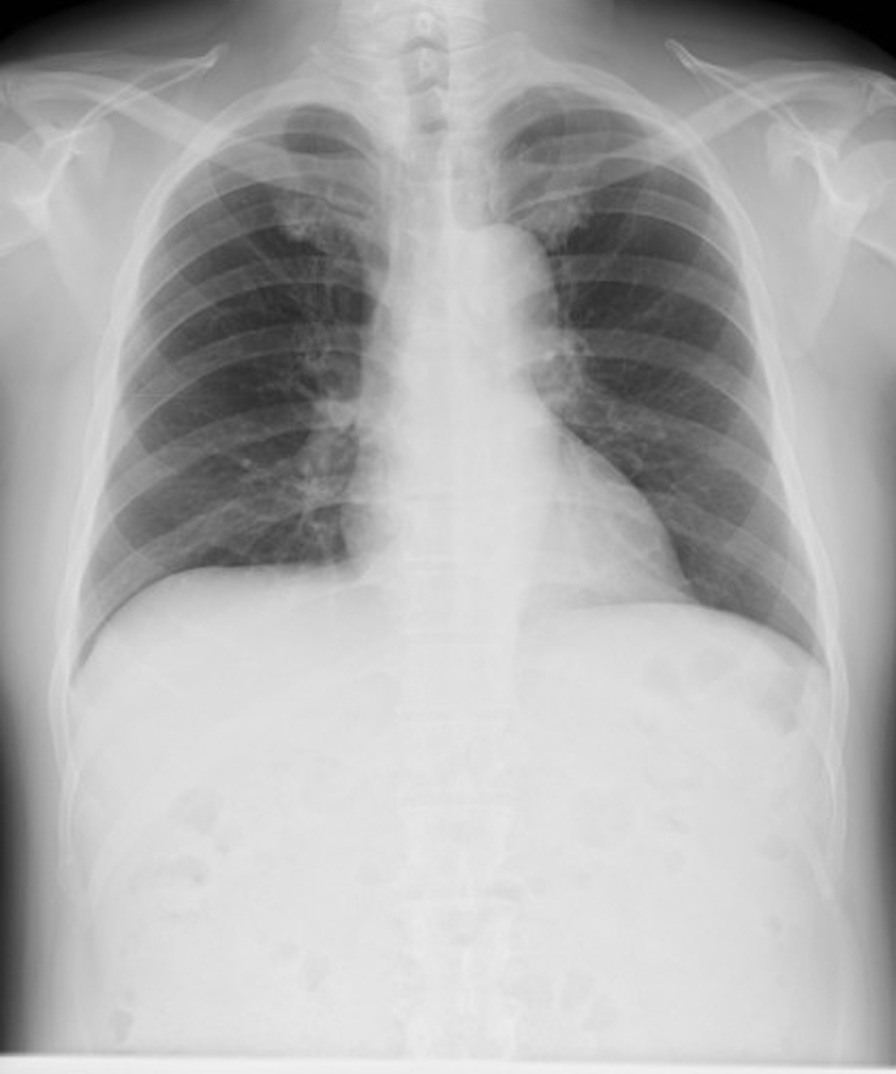
Chest X-ray 4 months prior to myocardial infarction. Normal findings are obsreved

**Fig. 2:**
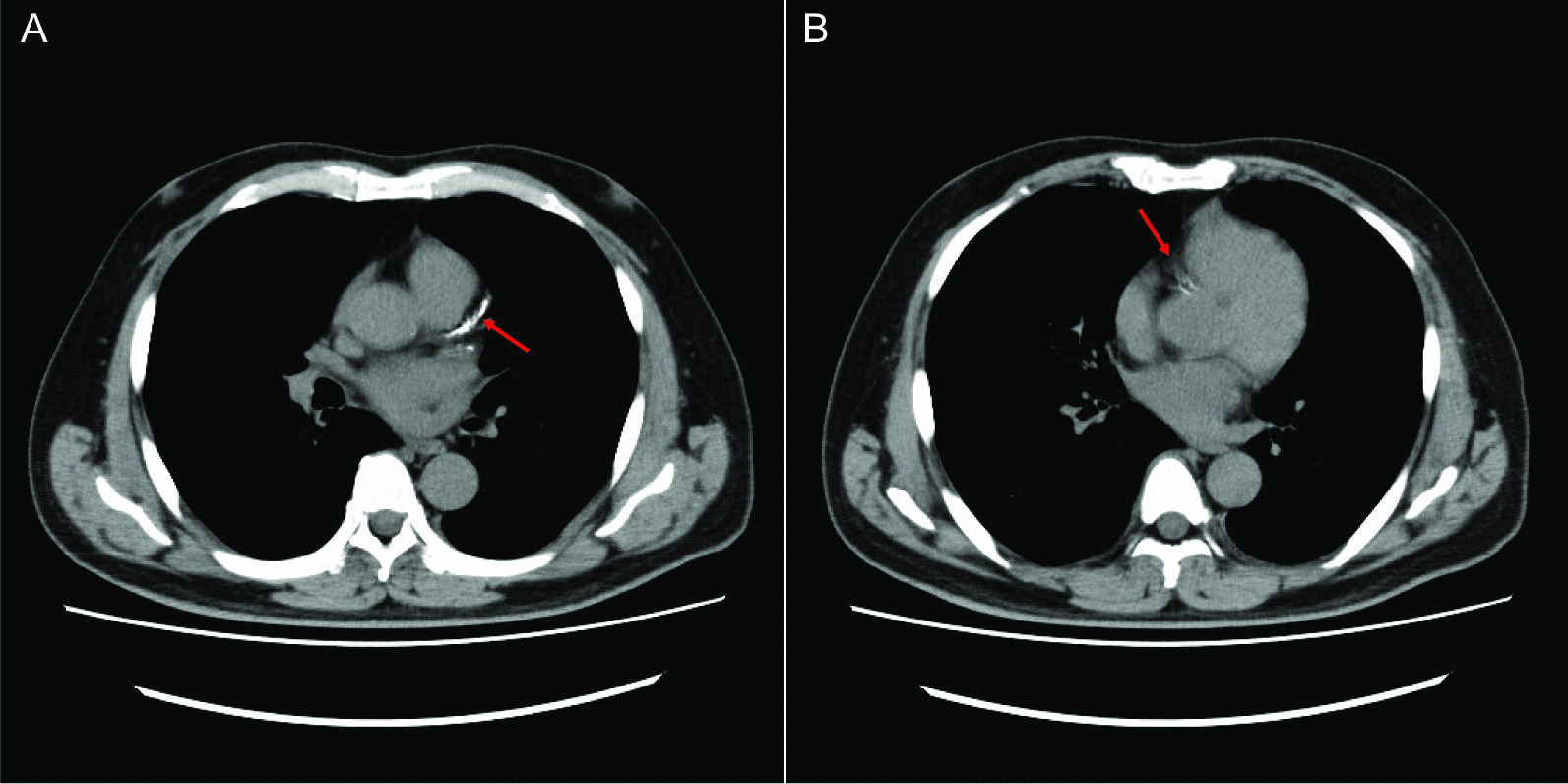
Computed tomography 5 months prior to myocardial infarction. **A** Calcification of the left anterior descending artery is seen (arrow). **B** Slight calcification of the right coronary artery is seen (arrow)

**Fig. 3:**
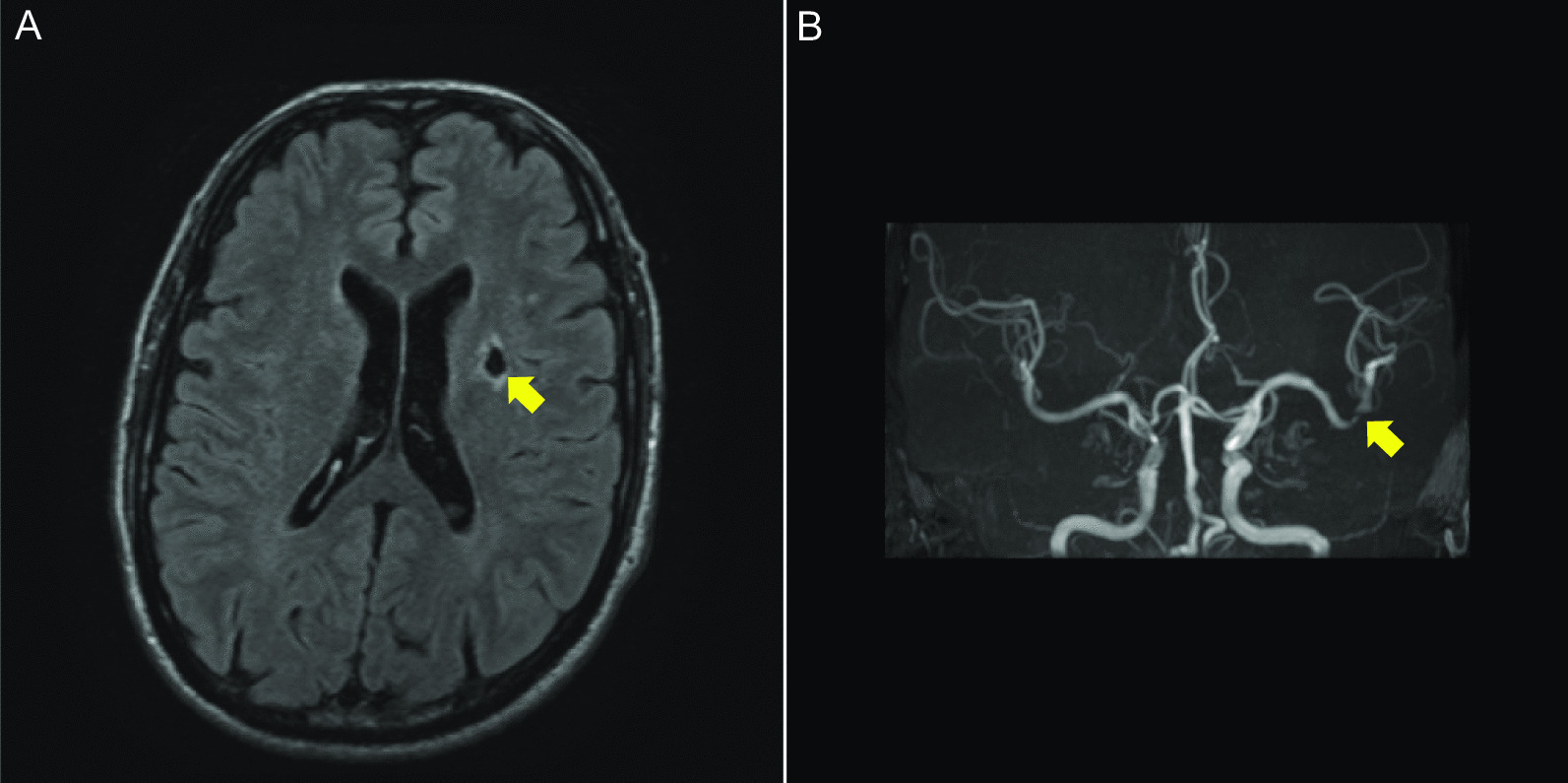
Magnetic resonance images of the brain 3 months prior to myocardial infarction. **A** Axial T2-weighted FLAIR MRI showing low intensity of the corona radiata (arrow). **B** Frontal view from magnetic resonance angiography showing stenosis of the left middle cerebral artery (arrow)

**Fig. 4:**
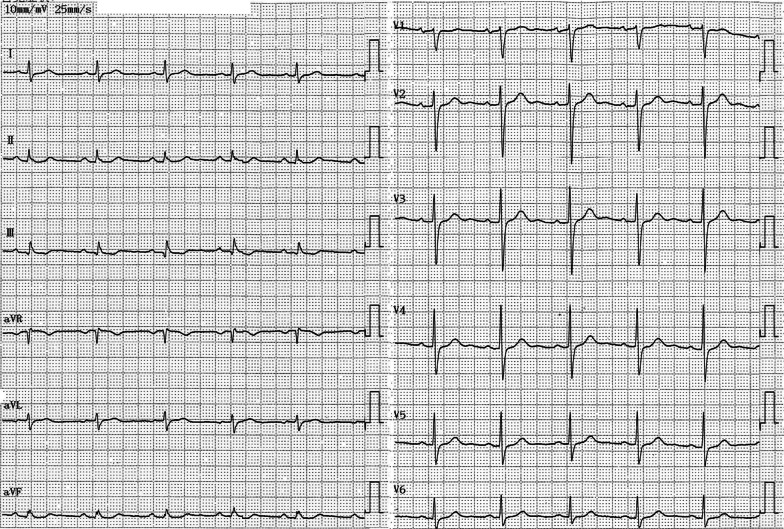
Electrocardiogram obtained before myocardial infarction. No ischemic changes are seen

**Fig. 5:**
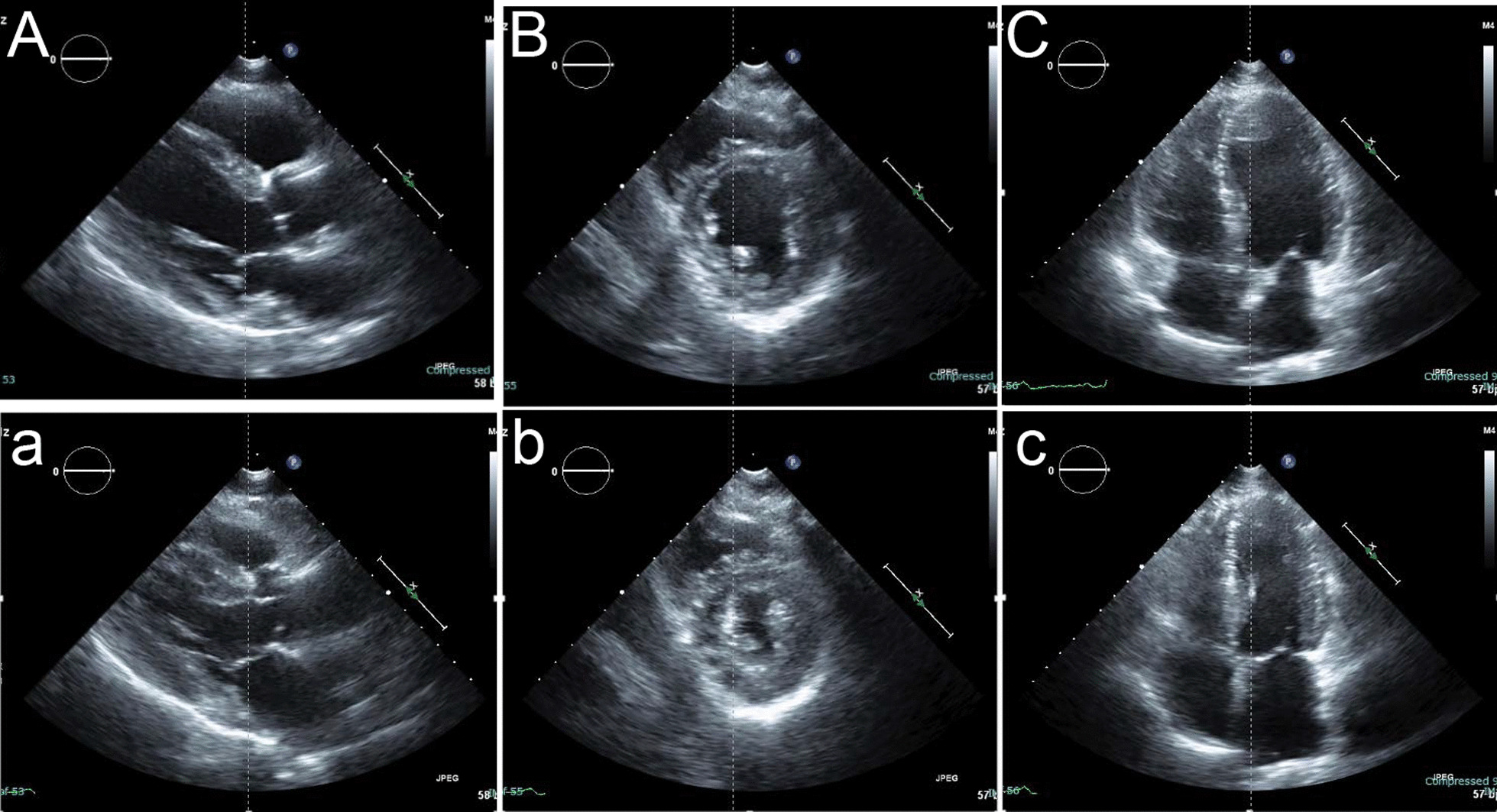
Echocardiogram obtained before myocardial infarction. Normal vetricular function is seen in the long-axis, short-axis, and four-chamber views in diastole (**A**, **B**, **C**, respectively) and systole (**a**, **b**, **c**, respectively)

**Fig. 6:**
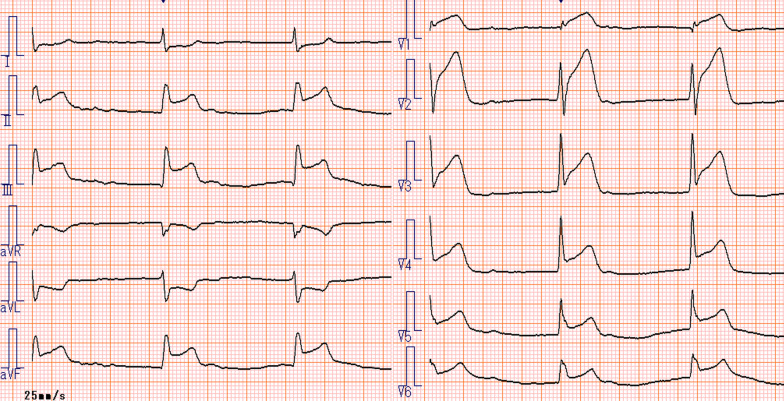
Electrocardiogram of the patient on arrival at the emergency room. Complete atrioventricular block; ST-segment elevation in leads V1-V6, II, III, and aVF; and ST-segment depression in leads I and aVL are seen

A 58-year-old man with hypertension, dyslipidemia, old cerebral infarction, and stage 4 chronic kidney disease had been an outpatient at our hospital’s nephrology department for 6 months poior to the cardiovascular event due to autosomal dominant polycystic kidney disease. He had no history of smoking or cardiovascular events. The initial work-up revealed normal findings in the chest X-ray (Fig. 1); calcification of the left anterior descending artery (LAD) and slight calcification of the right coronary artery (RCA) by computed tomography (Fig. 2); and cerebral infarction of corona radiata by brain magnetic resonance imaging (MRI); stenosis of the left middle cerebral artery by brain magnetic resonance angiography (MRA) (Fig. 3); and a serum creatinin level of 2.05 mg/dL by blood examination. ECG and echocardiography 3 months prior to the cardiovascular event had revealed normal findings (Figs. 4 and 5, respectively). Baseline echocardiography had shown no asynergy due to myocardial ischemia. After taking lunch, the patient felt severe crushing retrosternal chest pain, had one episode of vomiting, and called an ambulance. He reached our emergency department approximately 40 min after the onset of symptoms.He experienced cardiogenic shock (systolic blood pressure was 62 mmHg, diastolic blood pressure could not be measured, and heart rate was 44 beats/min) on arrival at the emergency department. ECG recorded 5 min after arrival revealed complete atrioventricular block (CAVB) and ST-segment elevation in leads II, III, aVF, and V1–V6 (Fig. 6). As a rule, we administer 200 mg of aspirin (loading dose) to patients with AMI at the emergency department, but it was not employed in this case due to the patient’s agony.

**Fig. 7:**
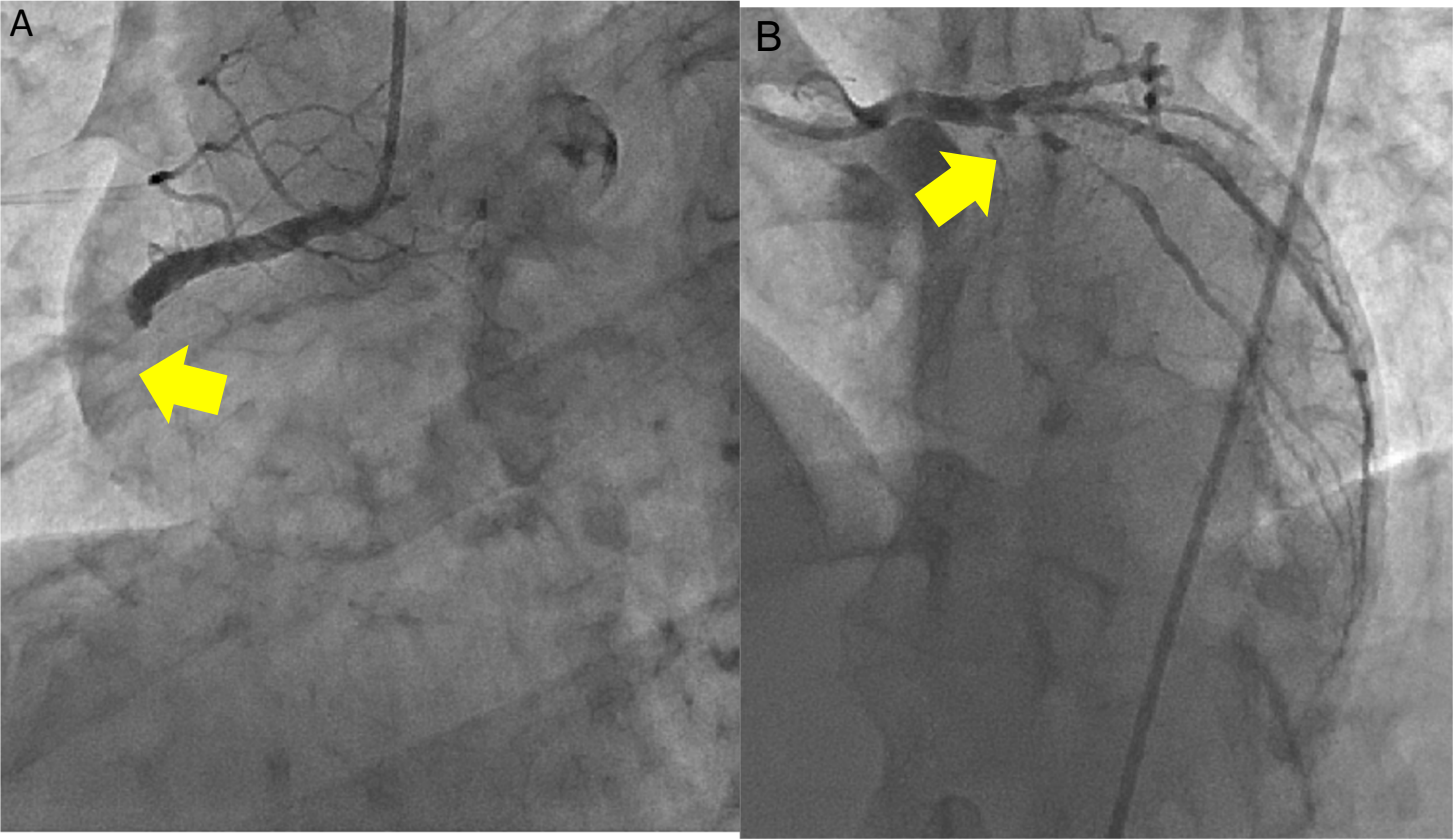
Emergency coronary angiography images. **A** Right coronary angiography image shows total occlusion of the proximal right coronary artery (arrow). **B** Left coronary angiography image shows total occlusion of the proximal left anterior descending artery (arrow)

**Fig. 8:**
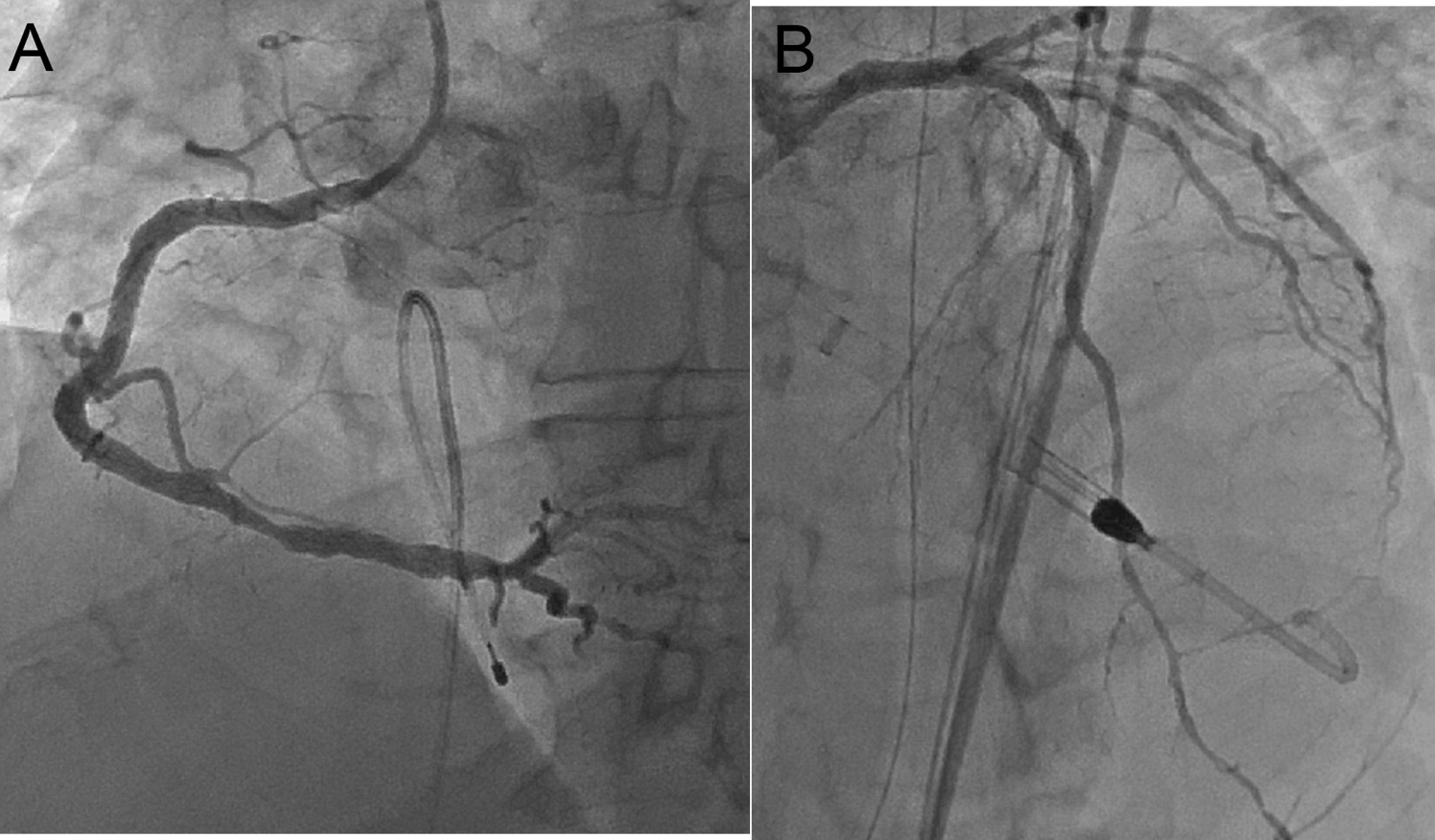
Post-stenting coronary angiography image. No residual stenosis and thrombolysis in myocardial infarction grade III recanalization are seen in the right coronary artery (**A**) and left anterior descending artery (**B**)

**Fig. 9:**
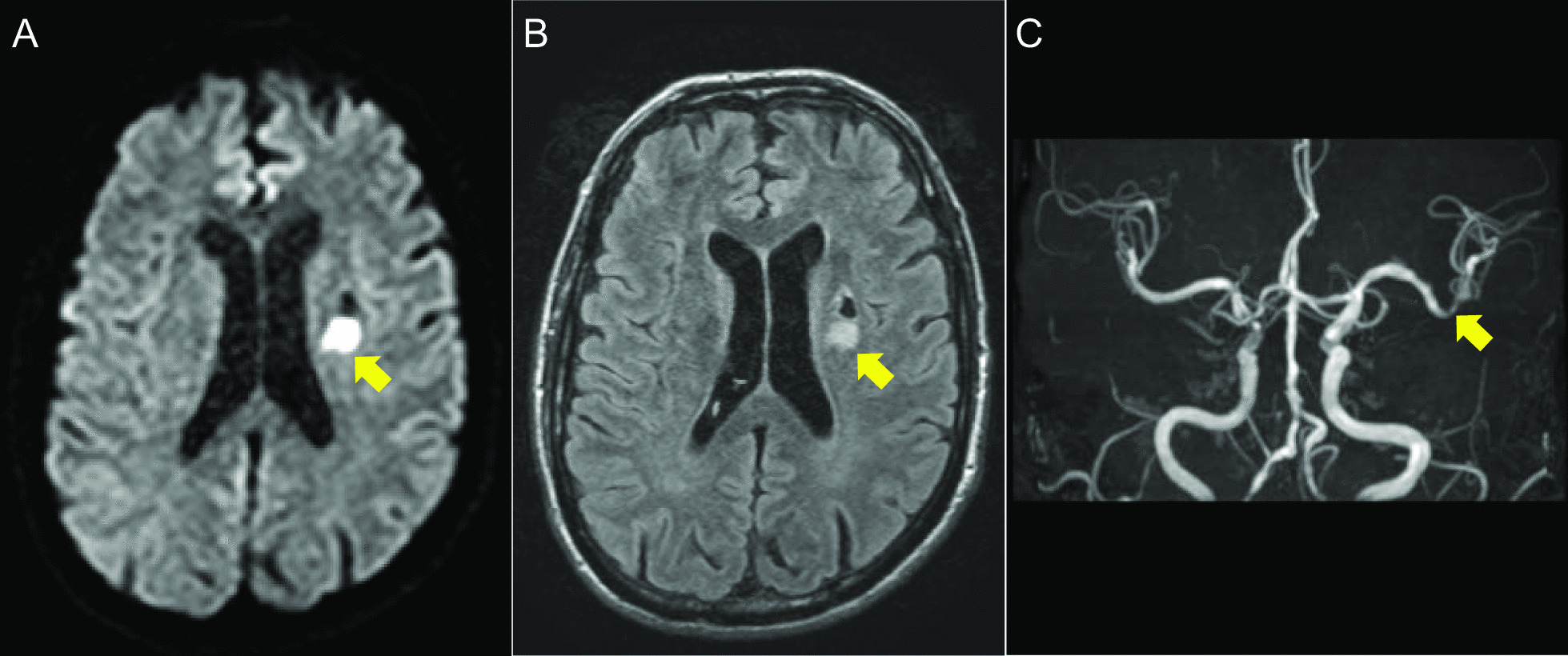
Magnetic resonance images of the brain after 8 days of removal of Impella device. **A** Axial diffusion-weighted MRI showing hyperintensity in the corona radiata (arrow). **B** Axial T2-weighted FLAIR MRI showing similar findings (arrow). **C** Frontal view from magnetic resonance angiography showing stenosis of the left middle cerebral artery (arrow)

Emergency coronary angiography (CAG) performed 30 min after arrival revealed total occlusion of the proximal LAD and proximal RCA (Fig. 7, Additional files 1 and 2), with no collateral flow to the LAD or RCA (Additional files 1 and 2). A transvenous cardiac pacing catheter was temporarily inserted, and the patient became hemodynamically stable. We decided to treat the RCA lesion before the LAD lesion; we considered the RCA to be the culprit vessel for AMI due to the presence of vomiting, which is a symptom specific to inferior myocardial infarction. A 0.014-inch floppy guidewire (ASAHI INTEC, Aichi, Japan) was easily passed through the RCA lesion, and red thrombi were aspirated. Thrombolysis in myocardial infaction (TIMI) grade II flow was confirmed by aspirating around the culprit lesion twice; therefore, we did not inject any thrombolytic drug into the RCA. After passing the Filtrap embolic protection filter (Nipro, Osaka, Japan), a 4.0 × 23 mm drug eluting stent (DES) was successfully deployed. TIMI grade III flow was observed in the RCA (Fig. 8A) and the CAVB disappeared. However, ST-segment elevation in precordial leads on ECG and the patient’s symptoms of shock persisted. Echocardiography showed hypokinesia of not only inferior but also anterior and septal walls and post-stenting angiography revealed no collateral flow from the RCA to the LAD. We then performed percutaneous coronary intervention (PCI) of the LAD with mechanical hemodynamic support from the Impella CP (Abiomed, Danvers, MA, USA). After tracheal intubation, we administered 200 mg of aspirin and 20 mg of prasugrel (loading dose) via the nasogastric tube. A 0.014-inch floppy guidewire (ASAHI INTEC, Aichi, Japan) was advanced through the LAD lesion. Intravascular ultrasound (IVUS) revealed no ruptured plaque and some low echoic area, which was suspected as a thrombus, in the culprit vessel. A paclitaxel-coated balloon and 2.75 × 38 mm DES were successfully deployed in the D1 and LAD, respectively, and TIMI grade III flow was achieved (Fig. 8B). PCI was completed approximately 3.5 h after the initiation of CAG. ST-segment elevation significantly decreased after LAD stenting, and the patient was transferred to our coronary care unit for further intensive care. The following were atarted the next day of the procedure: aspirin, 100 mg/day; prasugrel, 3.75 mg/day; atorvastatin, 20 mg/day; and lansoprazole, 15 mg/day. The Impella device was removed on the fourth postoperative day. The patient’s hemodynamic parameters were stable, and his inotropic requirement rapidly decreased. Renal function generally remained unchanged. Echocardiography revealed a mean ejection fraction of 48% with mild hypokinesia of the anterior, septal (certainly due to transient occlusion of the LAD), and inferior (certainly due to transient occlusion of the RCA) walls compared with the pre-AMI levels. However, right hemiparesis appeared, and MRI performed 8 days after Impella device removal revealed acute or subacute cerebral infarction of the left corona radiata (Fig. 9A and B). The patient continued receiving dual antiplatelet therapy. On the 57th day of hospitalization, he had New York Heart Association class II heart failure and was transferred to a rehabilitation hospital to promote post-stroke recovery of manual dexterity.

## Discussion and conclusions

Simultaneous thrombosis of multiple coronary arteries has been reported to occur in approximately 2.5% of patients undergoing primary PCI [2] and 1.3% of patients experience an AMI [3]. The low incidence may be because most patients experience sudden death due to ischemic heart disease before presenting to the hospital, and the incidence observed at autopsy of patients with sudden cardiac death is much higher (nearly 50%) [4]. We considered our patient to have simultaneous total occlusion of the LAD and RCA because ECG and echocardiography revealed extensive anterior-inferior myocardial infarction and angiography revealed no collateral flow from the RCA to the LAD after RCA stenting [5, 6].

Identifiable causes of simultaneous multi-vessel coronary thrombosis include coronary vasospasm, coronary embolism, concomitant plaque rupture, a hypercoagulable state due to malignancy or a genetic mutation, thrombocytosis, heparin-induced thrombocytopenia, idiopathic thrombocytopenic purpura, antithrombin III deficiency, hyperhomocysteinemia, hormonal therapy with tamoxifen, smoking, and cocaine abuse. No test results, comorbidities, past medical history, family history, or social history could be identified in this case to be associated with predisposing factors that could aggravate the patient’s condition. The following mechanisms have been postulated: (1) plaque rupture occurs either due to inflammation-mediated primary disruption of the fibrous cap or as a result of the extrinsic influence of sympathetic tone and catecholamine levels on intraluminal forces [7, 8]; (2) hemodynamic instability and hypotension due to the occlusion of one coronary artery results in blood stasis and acute occlusion of another artery with a severe underlying lesion [2]. We considered the latter mechanism to be relevant in this case because no ruptured plaque was identified at the LAD lesion site on IVUS. It has been suggested that impaired activity of the endothelial prostacyclin synthesizing system contributes to the formation of intra-arterial thrombi and that the thromboxane B_2_ concentration increases during the early stages of AMI. However, the underlying etiology remains unclear in other cases. Rupture of multiple plaques is considered the primary mechanism in cases in which there is no identifiable cause [9].

Mahmoud et al. [1] reported that in patients with STEMI, simultaneous thrombosis was more commonly observed in the RCA and LAD than in the RCA and left circumflex artery (LCX) or LAD and LCX, whereas ECG findings of ST-segment elevation were more commonly observed in the inferior leads alone than in both the inferior and anterior leads. 12-lead ECG is an important tool for diagnosis; it enables physicians to identify the culprit vessel and optimize treatment. However, the possibility of occlusion of the dominant LAD, which perfuses the inferior wall surrounding the cardiac apex, or a single coronary artery that perfuses both the anterior and inferior walls must be considered when ST-segment elevation in the inferior and anterior leads is noted on 12-lead ECG. Apparent ST-segment changes may not be observed on ECG in patients with AMI with simultaneous total occlusion of two coronary arteries due to intraventricular conduction disturbance after ventricular defibrillation and resuscitation. Swift and accurate ECG interpretation is important. It is not uncommon for patients with chronic total occlusion (CTO) in one coronary artery to develop another vessel occlusion suddenly with AMI. In most cases, physicians perform PCI to the culprit vessel only and treat the CTO lesion later. However, it must be avoided because misinterptretin the evidence of abrupt cutoff of a vessel as CTO may lead to sudden succumbing to AMI in such cases. It is important to suspect the presence of one more culprit vessel if ST-segment elevation on ECG cannot be resolved, patient’s symptom persist, hemodynamics unstability continues, and post-PCI angiography reveals no collateral flow to the lesion considered to be the CTO, as happened in our case.

Most patients with simultaneous total occlusion of multiple coronary arteries experience hemodynamic instability, with 28% experiencing cardiogenic shock, 22% experiencing life-threatening ventricular arrhythmias, and 22% requiring intra-aortic balloon pump (IABP) insertion [2]. A study reported that 41% of patients with simultaneous total occlusion of multiple coronary arteries presented with cardiogenic shock, 18% presented with bradyarrhythmia, and 38% underwent IABP insertion [1]. Reports exist of patients with multi-vessel myocardial infarction who underwent IABP insertion [5, 10, 11], and there is a report of a patient in whom neither IABP nor an Impella device were inserted [12]. Nevertheless, all previous reports emphasize the importance of early, aggressive, and efficient percutaneous revascularization. We assumed that the CAVB in our patient resulted from AMI and that it would improve through quick restoration of coronary flow. Indeed, the conduction disturbance resolved shortly after the patient underwent primary PCI for RCA. It has been previously reported that Impella LP2.5 insertion provides more effective and superior hemodynamic support than standard treatment with IABP counterpulsation in patients with cardiogenic shock caused by myocardial infarction [13]. Another study found that rapid door-to-support times and improved survival can be achieved through early mechanical support with an Impella device and invasive hemodynamic monitoring in patients presenting with acute myocardial infarction complicated by cardiogenic shock (AMI-CS) [14]. A case report highlighted that the Impella 2.5L can be applied to provide good mechanical hemodynamic support to facilitate early revascularization and may be useful for the treatment of cardiogenic shock and arrest [15]. Studies of patients with AMI-CS indicate that there was no significant difference between the effect of hemodynamic support with the Impella device and IABP on 30-day mortality [16]. O’Neill et al. [17] suggest that hemodynamic support with the Impella 2.5L provided prior to PCI is associated with more complete revascularization and better survival than that provided post-PCI in patients with refractory AMI-CS. In our patient, we performed primary PCI to achieve swift reperfusion of the ischemic inferior wall, as the advantages of this outweighed those of first placing an Impella device, which would require arterial access and delay the PCI. To the best of our knowledge, this is the first case of AMI caused by acute occlusion of two epicardial arteries in which Impella device implantation was performed as a part of treatment. Multi-vessel myocardial infarction is associated with a high mortality and complicated course of hospitalization. Our patient experienced a cerebral infarction. We presume that this stroke occured due to lacunar infarction or branch atheromatous disease because MRA revealed no occlusion of the middle cerebral artery (Fig. 6C), echocardiography revealed no intraventricular thrombus, and atrial fibrillation had not been documented. We strongly suspected atherosclerotic change rather than hypercoagulation state due to the use of Impella CP. A combination of judicious medical treatment, efficient primary PCI, and early use of mechanical support devices is crucial to improve the survival rate of patients with this disease, which has a high mortality rate.

Acute double-vessel coronary thrombosis is a serious event with a rapid and fatal course and poor prognosis. Prompt diagnosis and management are required to prevent complications such as cardiogenic shock and ventricular arrhythmias. The prognosis of patients with this condition can be improved through aggressive reperfusion therapy and mechanical support.

## Supplementary Information

**Additional file 1.** Emergency left coronary angiography. Emergency left coronary angiography showing total occlusion of the proximal left anterior descending artery with no collateral flow to the right coronary artery.

**Additional file 2.** Emergency right coronary angiography. Emergency right coronary angiography showing total occlusion of the proximal right coronary artery with no collateral flow to the left coronary artery.

## Data Availability

All data generated or analyzed during this study are included in this published article.

## Abbreviations

AMI: Acute myocardial infarction
STEMI: ST-elevation myocardial infarction
ECG: Electrocardiography
LAD: Left anterior descending artery
RCA: Right coronary artery
MRI: Magnetic resonance imaging
MRA: Magnetic resonance angiography
CAVB: Complete atrioventricular block
CAG: Coronary angiography
TIMI: Thrombolysis in myocardial infarction
DES: Drug eluting stent
PCI: Percutaneous coronary intervention
IVUS: Intravascular ultrasound
LCX: Left circumflex artery
CTO: Chronic total occlusion
IABP: Intra-aortic balloon pump
AMI-CS: Acute myocardial infarction complicated by cardiogenic shock

## References

[1] Mahmoud A, Saad M, Elgendy IY (2015). Simultaneous multi-vessel coronary thrombosis in patients with ST-elevation myocardial infarction: a systematic review. Cardiovasc Revasc Med.

[2] Pollak PM, Parikh SV, Kizilgul M, Keeley EC (2009). Multiple culprit arteries in patients with ST segment elevation myocardial infarction referred for primary percutaneous coronary intervention. Am J Cardiol.

[3] Maagh P, Wickenbrock I, Schrage MO, Trappe HJ, Meissner A (2008). Acute simultaneous proximal occlusion of two major coronary arteries in acute myocardial infarction: Successful treatment with percutaneous coronary intervention. J Interv Cardiol.

[4] Davies MJ, Thomas A (1984). Thrombosis and acute coronary-artery lesions in sudden cardiac ischemic death. N Engl J Med.

[5] Sia SK, Huang CN, Ueng KC, Wu YL, Chan KC (2008). Double vessel acute myocardial infarction showing simultaneous total occlusion of left anterior descending artery and right coronary artery. Circ J.

[6] Garbo R, Steffenino G, Dellavalle A, Russo P, Meinardi F (2000). Myocardial infarction with acute thrombosis of multiple major coronary arteries: a clinical and angiographic observation in four patients. Ital Heart J.

[7] Gertz SD, Roberts WC (1990). Hemodynamic shear force in rupture of coronary arterial atherosclerotic plaques. Am J Cardiol.

[8] Muller JE, Stone PH, Turi ZG, Rutherford JD, Czeisler CA, Parker C (1985). Circadian variation in the frequency of onset of acute myocardial infarction. N Engl J Med.

[9] Meltser H, Bhakta D, Kalaria V (2004). Multivessel coronary thrombosis secondary to cocaine use successfully treated with multivessel primary angioplasty. Int J Cardiovasc Interv.

[10] Lee WH, Hsu PC, Lin TH, Su HM, Lai WT, Sheu SH (2010). Acute myocardial infarction with simultaneous involvement of right coronary artery and left anterior descending artery: a case report. Kaohsiung J Med Sci.

[11] Miyauchi M, Izumo M, Akashi YJ, Suzuki K, Ryu S, Hashimoto N (2010). Acute myocardial infarction in a young adult patient with simultaneous total occlusion of double coronary arteries. J Echocardiogr.

[12] Nordkin I, Goldberg A, Israeli Z, Halabi M (2020). Complicated acute myocardial infarction with simultaneous occlusion of two coronary arteries. Clin Case Rep.

[13] Seyfarth M, Sibbing D, Bauer I, Fröhlich G, Bott-Flügel L, Byrne R (2008). A randomized clinical trial to evaluate the safety and efficacy of a percutaneous left ventricular assist device versus intra-aortic balloon pumping for treatment of cardiogenic shock caused by myocardial infarction. J Am Coll Cardiol.

[14] Basir MB, Schreiber T, Dixon S, Alaswad K, Patel K, Almany S (2018). Feasibility of early mechanical circulatory support in acute myocardial infarction complicated by cardiogenic shock: the Detroit cardiogenic shock initiative. Catheter Cardiovasc Interv.

[15] Asrress KN, Marciniak M, Briceno N, Perera D (2017). Cardiac arrest in acute myocardial infarction: concept of circulatory support with mechanical chest compression and Impella to facilitate percutaneous coronary intervention. Heart Lung Circ.

[16] Schrage B, Ibrahim K, Loehn T, Werner N, Sinning JM, Pappalardo F (2019). Impella support for acute myocardial infarction complicated by cardiogenic shock. Circulation.

[17] O’Neill WW, Schreiber T, Wohns DHW, Rihal C, Naidu SS, Civitello AB (2014). The current use of Impella 2.5 in acute myocardial infarction complicated by cardiogenic shock: results from the USpella Registry. J Interv Cardiol.

